# Association between Media Use and Bedtime Delays in Young Children: An Adjunct Study of the Japan Environment and Children’s Study

**DOI:** 10.3390/ijerph19159464

**Published:** 2022-08-02

**Authors:** Midori Yamamoto, Hidetoshi Mezawa, Kenichi Sakurai, Chisato Mori

**Affiliations:** 1 Department of Sustainable Health Science, Center for Preventive Medical Sciences, Chiba University, 1-33 Yayoi-cho, Inage-ku, Chiba 263-8522, Japan; cmori@faculty.chiba-u.jp; 2 Medical Support Center for Japan Environment and Children’s Study, National Center for Child Health and Development, 10-1, Okura 2-chome, Setagaya-ku, Tokyo 157-8535, Japan; mezawa-h@ncchd.go.jp; 3 Department of Nutrition and Metabolic Medicine, Center for Preventive Medical Sciences, Chiba University, 1-33 Yayoi-cho, Inage-ku, Chiba 263-8522, Japan; sakuraik@faculty.chiba-u.jp; 4 Department of Bioenvironmental Medicine, Graduate School of Medicine, Chiba University, 1-8-1 Inohana, Chuo-ku, Chiba 260-8670, Japan

**Keywords:** delayed bedtime, screen media, screen time, multi-device, young children

## Abstract

Excessive screen media use has been reported to cause shorter sleep; however, the types of media environments that affect early childhood sleep are less known. This study examined the association of multiple media use, screen time for each device, and the purpose of smartphone and tablet use with delayed bedtime among 4–8-year-olds. Participants were recruited from the Japan Environment and Children’s Study, a nationwide birth cohort study. Mothers of 1837 children reported screen media use and bedtime in a questionnaire. The association between delayed bedtimes (after 22:00 h) and media device use (smartphones, tablets, portable and console games, and TV/DVDs) was examined by logistic regression analysis. Children who used three or more devices besides TV/DVDs were more likely to have delayed bedtimes. Delayed bedtimes were associated with smartphone use, even with a 0.1–1 h/day screen time, and with prolonged screen time for tablets, portable games, and console games, but not for TV/DVDs. Gaming on smartphones and tablets was also associated with delayed bedtime. To ensure adequate sleep for young children, families must develop feasible measures to discourage children’s use of multiple devices and prolonged device use, especially for games, and a social environment that supports such measures.

## 1. Introduction

The use of various screen media is rapidly expanding worldwide. Children are increasingly exposed to screen media devices from an early age [1,2,3]. According to a 2017 report from Japan, smartphones, tablets, portable games, and console games were the most popular devices among children aged 0–9, with 3.1% of children less than 1 year old, 55.2% of 5-year-olds, and 89.9% of 9-year-olds using devices with Internet access [3]. The American Academy of Pediatrics (AAP) recommends limiting screen use for children aged 2–5 years to high-quality programs for 1 h per day [4]. However, many children use screen media for much longer durations [1,2,3]. While screen media devices with Internet access are a useful tool for acquiring and communicating information, there are grave concerns about the undesirable health effects of screen media use on children and adolescents [5,6].

One of the health-related effects of screen media use is reduced sleep duration. Studies have consistently shown an association between screen media use and later bedtime or shorter total sleep time in school-aged children and adolescents [7,8,9]. In particular, screen media use before bedtime has been reported to be associated with delayed bedtime and shorter total sleep time [9]. Furthermore, late bedtimes and shorter sleep duration have been highlighted as important factors in early childhood development; they have also been shown to be associated with obesity and other adverse health outcomes [10,11,12].

Early childhood is a critical period for forming long-term habits in later life [13] and is more vulnerable to environmental health effects than the period of adolescence. In today’s social environment of widespread and diverse media use, young children are increasingly exposed to a variety of media devices. Studies have shown an association between excessive media use, such as smartphones, video games, TV, or gaming, and shorter sleep time [14,15,16,17], heavier TV or tablet use and later bedtimes [18], and a longitudinal association between computer use or TV viewing and shorter sleep duration [19]. In the Japan Environment and Children’s Study (JECS), prolonged screen time of TV/DVD or smartphone/portable electronic device was associated with delayed bedtime in children at the age of 3 years [20]. However, in modern society, it is important to evaluate the impact of individual and multiple devices.

Mobile screen media use and sleeping behavior in young children may be affected by accessibility to media devices at home [21]. Regarding the multiple screen media used by young children, identifying the uses associated with delayed bedtime can provide important information to help families and caregivers understand the aspects that need attention. Therefore, this study aimed to clarify the kind of media environments in early childhood that are associated with delayed bedtimes among four- to eight-year-old Japanese children from three perspectives: First, we determined if multiple device use was associated with delayed bedtime. Second, we investigated the associated devices. Finally, we identified the purpose of device use.

## 2. Materials and Methods

### 2.1. Study Population

This study was conducted as an adjunct study of the JECS, which was a nationwide government-funded birth cohort study that aimed to evaluate the impact of environmental factors on children’s health and development. A total of 97,415 pregnant women (103,062 pregnancies) were registered at 15 regional centers located throughout Japan between January 2011 and March 2014. Details of the JECS can be found in a study by Kawamoto et al. [22].

All mothers enrolled in the JECS at the Chiba Regional Center as of December 2018 were invited to participate in this adjunct study and voluntarily completed a questionnaire. If more than one child per household participated in the JECS, data pertaining to the oldest child were used. In this adjunct study, informed consent was not obtained, but the opportunity to refuse was ensured by indicating that responses to the questionnaire were voluntary. This study was approved by the Research Ethics Committee of the Graduate School of Medicine, Chiba University (3028).

### 2.2. Data Collection

The questionnaire developed for this study collected data related to the following: (i) children’s regular bedtime, (ii) children’s media device use and screen time, (iii) frequency of children’s screen media use within 1 h before sleeping, (iv) Internet content accessed on smartphones and tablets, (v) parental screen time for mobile devices, (vi) basic parental characteristics (age, educational level, household income, and mother’s occupation), and (vii) family structure. Missing data on parental age and educational level were complemented using the JECS dataset. The following information was also extracted from the JECS dataset: child’s sex, birth year, elder sibling, household income, mother’s occupation and marital status, child’s diagnosis of autism spectrum disorder by age 4, and mother’s and child’s screen media use at 3 years of age. The specific JECS datasets used in this study were “jecs-an-20180131” released in March 2018 and “jecs-qa-20210401” released in February 2022.

### 2.3. Measures

#### 2.3.1. Outcome

Children’s delayed bedtime was the primary outcome measure. Mothers were asked to report their children’s regular bedtime by the following question: “What time does your child usually go to bed at night?” The responses were collected from among five categories (before 19:59, 20:00–20:59, 21:00–21:59, 22:00–22:59, and after 23:00 h). Delayed bedtime was defined as 22:00 h or later. This was based on the reported average bedtime of 21:29 h for Japanese infants and toddlers [23] and the fact that studies of children and Japanese survey reports set the cutoff point for delayed bedtime at 22:00 h [24,25,26].

#### 2.3.2. Exposure

For child’s screen media use as exposure, the following strategies were used in the analysis. First, we examined whether single-device and multi-device use of each of the four devices (smartphone, tablet, portable game, and console game) was associated with delayed bedtime. TV/DVD was not included in the analysis because it was used by nearly 100% of the children (97.0%). Second, we investigated the screen time for which device was strongly associated with delayed bedtime. Third, for smartphones and tablets that allowed access to multiple contents, we examined which uses were associated with delayed bedtimes.

Device use and screen time were investigated by the following questions: “Please indicate the amount of time your child uses the following devices (smartphones, electronic tablets, portable game devices, console game devices, TV/DVDs, and personal computers) on a typical weekday and on weekends.” The devices were selected based on survey reports on children’s media use [1,2,3]. The responses were collected using seven categories (0, 0.1–0.9, 1.0–1.9, 2.0–2.9, 3.0–3.9, 4.0–4.9, and ≥5 h) and converted to corresponding numbers ranging from “0 h” to “5.5 h.” Time used for studying at school, cram school, and so forth, was excluded. Daily screen time on each device was calculated as the average of 5 weekdays and 2 weekends. Screen time on weekdays and weekends was also calculated as an average. Total screen time was calculated for four devices: smartphones, tablets, portable game devices, and console game devices. The total screen time including TV/DVD use was not analyzed because of a small number of children in the “none (0 h/day)” group (14 cases). Use of a personal computer was not included in the analysis as few children used it.

#### 2.3.3. Covariates

Covariates were selected a priori based on the literature [9,17,21] and biological plausibility. The adjusted model included child’s sex (male/female), age (4, 5, 6, and 7–8 years), presence of elder sibling (no/yes), parents’ age (<40 and ≥40 years), parents’ education (<13, 13–15, and ≥16 years), household income (<4 million, 4–5.9 million, and ≥6 million Japanese yen), mother’s occupation (no/yes), and screen media use within 1 h before sleeping (<1, 1–3, and ≥4 times/week). In an analysis of the association between delayed bedtime and child’s screen time for each device (smartphone, tablet, portable game, console game, and TV/DVD), the adjusted model also included screen time for other devices. In addition to the adjusted model, parents’ screen time for mobile devices (≤1, 1.1–2, 2.1–3, and >3 h/day) was included as Adjusted Model 2. Daily screen time for parents’ mobile devices was calculated in the same way as for children’s screen time.

### 2.4. Statistical Analysis

The characteristics of the participants of this study and other JECS participant mothers were compared using the JECS datasets. Pearson’s chi-squared test or Fisher’s exact test for categorical variables and Mann–Whitney U test for continuous variables were used to test for between-group differences. Multivariable binomial logistic regression analyses were performed to examine the association between screen media use and delayed bedtime. The missing values for covariates were complemented by multiple imputations. In the process, each missing value was replaced with a series of substituted plausible values by creating 10 filled-in complete datasets using exposures, an outcome, and covariates.

We performed two sensitivity analyses to assess the robustness of the findings. (1) Complete case analyses were performed by excluding missing data for covariates. (2) Diagnosis of autism spectrum disorder (no/yes) for the child was added to Adjusted Model 2 for the association between screen time and delayed bedtime. All analyses were conducted using SPSS ver. 27 (IBM Corp., Armonk, NY, USA).

## 3. Results

### 3.1. Characteristics of Respondents

Of the 5068 mothers who participated in the JECS at the Chiba Regional Centre, 1909 volunteered for this adjunct study, and 1837 were eligible respondents with complete data for bedtime and screen media use. The eligible respondents were more likely to have slightly higher parental age, higher education and income, and lesser media use among mothers and children when the child was 3 years old. Other characteristics of eligible respondents were similar to the other participants of the JECS (Table S1).

**Figure 1:**
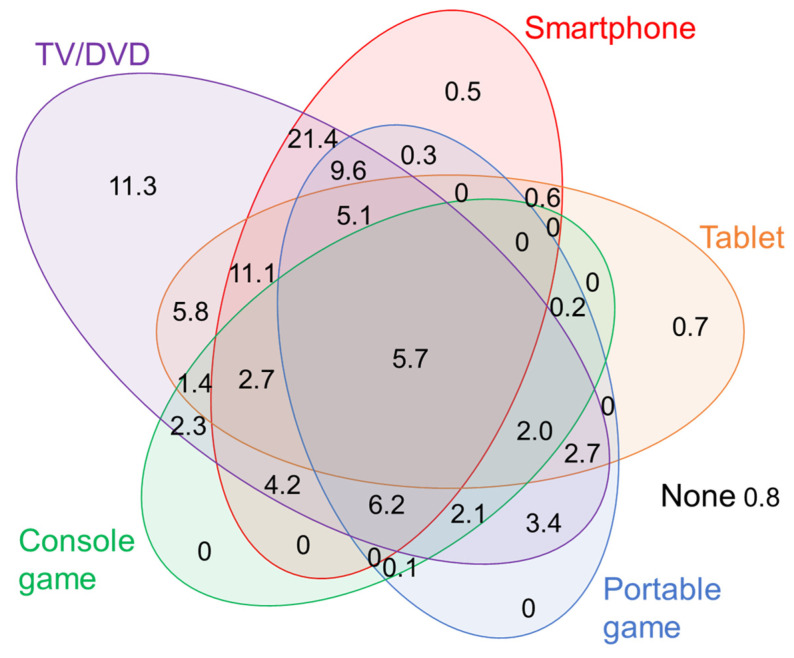
Venn diagram of children’s media use. Values are in %.

Table 1 shows the characteristics of the eligible respondents. Among children, 51.2% were boys. The children’s ages ranged from 4 years to 8 years and 2 months. More than half of the children went to bed between 21:00 and 21:59 h, and 11.4% after 22:00 h. Most children used TV/DVD (97.0%). For other devices, 67.3% of the children used smartphones, 37.8% used tablets, 37.3% used portable games, and 26.8% used console games. Children’s media device use was displayed in a Venn diagram to visualize the intersection of multi-device use (Figure 1). Among children in the eligible respondents, 11.3% used only TV/DVD, 85.6% used TV/DVD and any devices other than TV/DVD, and 2.3% used only devices other than TV/DVD.

### 3.2. Association between Single- or Multi-Device Use and Children’s Delayed Bedtime

We examined the association between the number of devices used and delayed bedtime for smartphones, tablets, portable games, and console games (Table 2). Among all children, 222 (12.1%) were non-device users (0.8% did not use any device and 11.3% used only TV/DVD), 627 (34.1%) used only one device (apart from TV/DVD), and 988 (53.8%) used two or more devices. For all four devices, multi-device users were more likely to delay bedtime compared with non-device users, with adjusted odds ratios (aOR) [95% confidence interval (CI)] of 1.98 [1.12–3.50] for smartphone and other device users, 2.08 [1.16–3.71] for tablet and other device users, 2.09 [1.16–3.76] for portable game and other device users, and 2.32 [1.27–4.24] for console game and other device users. Smartphone-only users also tended to delay bedtime. Children who used three or four devices had a higher risk of delayed bedtime, with aOR [95% CI] of 2.31 [1.23–4.34] for three devices and 4.33 [2.11–8.90] for four devices.

### 3.3. Association between Screen Time and Children’s Delayed Bedtime

For smartphones, a screen time of less than 1 h/day was associated with delayed bedtime (aOR [95% CI], 1.85 [1.25–2.75]), and longer screen time showed a stronger association with delayed bedtime (4.94 [2.85–8.55] for 1.1–2 h/day and 3.70 [1.73–7.87] for >2 h/day) (Table 3). Delayed bedtime was also associated with more than 1 h/day of screen time for portable games (2.33 [1.29–4.20] for 1.1–2 h/day and 2.87 [1.24–6.61] for >2 h/day) and with more than 2 h/day screen time for tablets (2.88 [1.63–5.09]) and console games (4.04 [1.21–13.44]). Conversely, a screen time of 2 h/day or less for TV/DVD was negatively associated with delayed bedtime (0.35 [0.16–0.81] for 0.1–1 h/day and 0.44 [0.20–0.96] for 1.1–2 h/day). Increased total screen time was associated with delayed bedtime (2.30 [1.25–4.24] for 1.1–2 h/day, 3.66 [1.69–7.90] for 3.1–4 h/day, and 8.57 [4.16–17.65] for >4 h/day). When parents’ mobile device screen time was additionally adjusted (Adjusted Model 2), the odds ratios for smartphones, tablets, and portable games changed little. In contrast, the odds ratio for console games with screen time of more than 2 h/day increased.

When screen time was divided into weekdays and weekends, the odds ratio for delayed bedtime increased as the total screen time increased from more than 1 h/day on both days. Overall, children spent less time using media devices on weekdays than on the weekend. However, those who spent a long time using devices on weekdays were more likely to have delayed bedtime (more than 1 h/day for smartphones and portable games, more than 2 h for tablets and console games, and more than 2 h/day of total screen time) (Table 4).

### 3.4. Association between Internet Content and Children’s Delayed Bedtime

Among the kinds of Internet content accessed on smartphones and tablets, gaming was associated with delayed bedtime (aOR [95% CI] of 1.75 [1.16–2.65] for smartphones and 2.20 [1.31–3.69] for tablets). Intellectual training on smartphones was also associated with delayed bedtime (1.65 [1.05–2.60]). Smartphone users accessing three or more kinds of content were more likely to delay bedtime than users accessing only one kind of content (2.23 [1.34–3.70]) (Table 5).

### 3.5. Sensitivity Analysis

Two sensitivity analyses were performed, one for complete cases by excluding missing data for covariates (n = 1741), and the other by including doctor-diagnosed autism spectrum disorder (yes = 0.5%), in the adjusted model of the analysis of the association between children’s screen time and delayed bedtime. In both analyses, as in the main analysis, prolonged screen time for smartphones, tablets, portable games, and console games and their total screen time were positively associated with delayed bedtime, and screen time for TV/DVD was negatively associated with delayed bedtime.

## 4. Discussion

To identify the media environments that are associated with bedtime delay among Japanese children aged 4 to 8 years, we conducted analyses focusing on three key factors: (1) single or multiple device use, including smartphones, tablets, portable games, and console games; (2) screen time per device (including TV/DVD); and (3) Internet content accessed via smartphones and tablets.

Almost all children used TV/DVD (97.0%), but 85.6% of them also used either a smartphone, tablet, portable game, or console game, with a minority (11.3%) using only TV/DVD. Nowadays, even young children use multiple screen media, not just TV/DVDs [1,2,3]. Children who use only TV/DVDs possibly belong to families that strictly control their children’s media use.

Cultural differences may cause differences in bedtime [23,24]. Mindell et al. reported that bedtimes for infants and toddlers in 17 Asian and Caucasian countries ranged from 19:27 to 22:17 h, with 21:29 h being the average in Japan [23]. This study defined delayed bedtime as 22:00 h or later. For four devices, excluding TV/DVD, children who used multiple devices were more likely to delay bedtime than non-device users (those who did not use any of the four devices). In addition, prolonged screen times for individual devices and prolonged total screen times were strongly associated with delayed bedtimes. These results suggest that the overall increase in exposure to these media devices contributed to delayed bedtime. Prolonged screen time on weekdays was particularly strongly associated with delayed bedtime. The associations persisted even after adjusting for risk factors such as children’s device use before sleeping and parental age and education, suggesting that young children’s media use itself was associated with delayed bedtime. While a systematic review has shown that parental device use is associated with increased screen time in children [21], the association between children’s screen time and delayed bedtime was either independent of parental device use or the risk increased after adjusting for parental screen time. Since even young children are likely to use media devices on their own [1,27], it is important to establish rules and create a disciplined environment for children’s media use at home [28].

Smartphones were quite popular among young children [2,3], with 67.3% of children in this study using them. This study showed that smartphone use by young children was remarkably associated with delayed bedtime compared to other devices. Not only multi-device users; but smartphone-only users also tended to delay bedtime. Compared with non-smartphone users, those who used a smartphone were more likely to delay bedtime even for short screen times of 0.1–1 h/day, and longer screen time was strongly associated with delayed bedtime. Children who accessed more types of content on their smartphones tended to delay bedtime; suggesting that smartphone dependence may be linked to later bedtimes.

It should be noted that playing games on any device was likely to lead to delayed bedtimes. Prolonged use of portable and console games was strongly associated with delayed bedtime, and children who used smartphones and tablets for gaming were more likely to delay bedtime. This could be due to the “addictive nature” of gaming [29]. Intellectual training was also found to be associated with delayed bedtime for smartphones. This may be because educational apps for young children are designed to be enjoyed as games. However, video sharing was the most popular use of smartphones and tablets but was not associated with delayed bedtimes.

Our results support other studies that have reported that smartphone/tablet use, and gaming, were related to shorter sleep or later bedtime in young children [14,15,16,17,18]. However, unlike previous findings [17,18], the present study showed a lesser delay in bedtime for children who viewed TV/DVDs than for those who did not. This may be attributed to the fact that considerably fewer children did not use TV/DVD, and among these children, a larger proportion (19.4%) had delayed bedtime. Some other activity other than TV/DVD viewing may have contributed to delayed bedtime. Alternatively, young children may rather watch TV/DVDs at night as a kind of sleep aid [30] and go to bed earlier. Moreover, it could be that children are not as focused on the TV screen as they are on their smartphones, tablets, or game consoles [20].

Several possible mechanisms can be proposed for the association of screen media use with delayed bedtime. First, time spent using media devices can directly delay bedtime [7]. Second, exposure to bright light from the screen at night may suppress the secretion of the sleep-promoting hormone melatonin, and thus, delay the circadian rhythm [31]. Melatonin suppression by exposure to bright light from self-luminous devices may last for 2 h [32]. Third, screen media use, especially gaming in the evenings, may cause physiological arousal [33].

The strength of this study is that it collected a wide range of information on children’s device use. However, this study also has several limitations. First, respondents who participated in this adjunct study and had complete data represented 36.2% of the mothers who participated in the JECS at the Chiba Regional Center. The participation rates of the adjunct study may have caused a non-response bias. Compared to eligible respondents, the other mothers who participated in the JECS had a slightly larger number of children with longer media use at 3 years of age. Therefore, non-response bias may contribute to underestimating the prevalence of prolonged device use. However, the bias may have little effect on the associations between outcomes and factors and is not likely to pose a threat to the validity of the results [34,35]. Second, screen time for media devices in this study was surveyed through a self-administered questionnaire completed by mothers. This method could conceivably underestimate actual screen time due to social desirability bias or parents’ lack of awareness of their children’s device use, but it could also overestimate [36]. Third, other leisure time, such as children’s outdoor play time, was not measured and therefore not adjusted for. In addition, the prolonged use of media devices may include factors related to the child’s atypical development [37,38]. In our sensitivity analysis, additional adjustment for autism diagnosed before age 4 resulted in little change in odds ratios. However, no adjustment was made for attention-deficit/hyperactivity disorder because data were not available by age 4. Fourth, because this study was conducted in suburban and rural areas for birth cohort participants, caution should be exercised in generalizing the study findings. Nevertheless, the prevalence of screen media use in young children in this study was similar to the results of a survey conducted by the Cabinet Office in Japan in 2018 [3]. Therefore, the present results seem to be representative to some extent. Finally, children’s bedtime, device use, and other factors were examined only cross-sectionally; therefore, their causal relationships could not be clarified. Longitudinal studies are needed to investigate these causal relationships.

The media environment has changed markedly and is expected to continue to change. The COVID-19 pandemic has expanded the use of screen media in early childhood [39]. Creating an environment for the appropriate use of media devices by young children requires an approach that is tailored to each family’s situation. Social support tools, such as the American Academy of Pediatrics recommendations [4], may be necessary to help children and their families develop feasible strategies to determine which media to use and how. Children’s prolonged media use may not be controlled by a parental approach alone. There may be socioeconomic or environmental factors (such as having only a few playgrounds) that lead to children and their families spending a longer time on media [40], and future research should take these factors into account.

## 5. Conclusions

This study found that multi-device use, prolonged screen time, and smartphone use and gaming were associated with delayed bedtime in early childhood. In order to ensure adequate sleep for young children, a home environment with appropriate media device use is necessary, and school educators and health care providers may need to support each family to address this in a feasible manner.

## Figures and Tables

**Table 1:** Characteristics of children and parents.

	N or Mean	% or (SD)
	1837	
*Basic characteristics*		
Child’s sex		
Male	940	51.2
Female	897	48.8
Child’s age, years		
4	432	23.5
5	616	33.5
6	626	34.1
7–8	163	8.9
Elder sibling		
0	822	44.7
≥1	1015	55.3
Mother’s current age, years		
mean (SD)	37.6	(4.8)
Father’s current age, years		
mean (SD)	39.5	(5.9)
Missing	5.0	0.3
Mother’s education		
Junior or senior high school	553	30.1
Junior college or vocational	810	44.1
Undergraduate or above	466	25.4
Missing	8	0.4
Father’s education		
Junior or senior high school	694	37.8
Junior college or vocational	436	23.7
Undergraduate or above	694	37.8
Missing	13	0.7
Household income, million Japanese Yen		
<4	552	30.0
4 to <6	631	34.3
≥6	587	32.0
Missing	67	3.6
Mother’s occupation		
No	513	27.9
Yes	1309	71.3
Missing	15	0.8
Mother’s marital status		
Married	1735	94.4
Single	102	5.6
*Child’s screen media use within 1 h before sleeping*		
<1 time/week	388	21.1
1–3 times/week	489	26.6
4–7 times/week	955	52.0
Missing	5	0.3
*Child’s media device use*		
Smartphone	1237	67.3
Tablet	694	37.8
Portable game	685	37.3
Console game	492	26.8
TV/DVD	1781	97.0
*Child’s regular bedtime*		
Before 19:59	66	3.6
20:00–20:59	552	30.0
21:00–21:59	1009	54.9
22:00–22:59	196	10.7
After 23:00	14	0.8

SD, standard deviation.

**Table 2:** Associations between children’s delayed bedtime (after 22:00 h) and use of media devices (N = 1837).

Use Of Media Device	n	% of Cases	Crude		Adjusted	
			aOR	[95% CI]	aOR	[95% CI]
Non-device users	222	7.2	1.00	reference	1.00	reference
*Smartphone*						
Smartphone-only users	403	12.7	**1.87**	**[1.04–3.36]**	1.68	[0.92–3.06]
Smartphone and other device users	834	14.0	**2.10**	**[1.22–3.62]**	**1.98**	**[1.12–3.50]**
*Tablet*						
Tablet-only users	119	5.9	0.80	[0.32–2.01]	0.68	[0.27–1.73]
Tablet and other device users	575	15.1	**2.30**	**[1.31–4.01]**	**2.08**	**[1.16–3.71]**
*Portable game*						
Portable game-only users	63	6.3	0.87	[0.28–2.71]	0.83	[0.26–2.64]
Portable game and other device users	622	13.8	**2.07**	**[1.18–3.61]**	**2.09**	**[1.16–3.76]**
*Console game*						
Console game-only users	42	2.4	0.31	[0.04–2.43]	0.42	[0.05–3.30]
Console game and other device users	450	14.7	**2.21**	**[1.25–3.92]**	**2.32**	**[1.27–4.24]**
*Number of devices used*						
None	222	7.2	1.00	reference	1.00	reference
1 device	627	10.0	1.44	[0.81–2.55]	1.32	[0.74–2.37]
2 devices	588	10.5	1.52	[0.86–2.69]	1.49	[0.82–2.69]
3 devices	295	14.9	**2.26**	**[1.24–4.12]**	**2.31**	**[1.23–4.34]**
4 devices	105	23.8	**4.02**	**[2.04–7.93]**	**4.33**	**[2.11–8.90]**

aOR: adjusted odds ratio; CI: confidence interval. The model included child’s sex, child’s age, presence of elder sibling, parents’ age, parents’ education, household income, mother’s occupation, and screen media use within 1 h before sleeping. Bold numbers indicate *p* < 0.05.

**Table 3:** Associations between children’s delayed bedtime (after 22:00 h) and screen time (N = 1837).

Screen Time	n	% of cases	Crude		Adjusted		Adjusted 2	
			aOR	[95% CI]	aOR	[95% CI]	aOR	[95% CI]
*Smartphone*								
None	600	7.0	1.00	reference	1.00	reference	1.00	reference
0.1–1 h/day	1055	11.2	**1.56**	**[1.08–2.27]**	**1.85**	**[1.25–2.75]**	**1.90**	**[1.28–2.82]**
1.1–2 h/day	125	28.0	**4.66**	**[2.80–7.75]**	**4.94**	**[2.85–8.55]**	**5.16**	**[2.96–9.00]**
>2 h/day	57	26.3	**3.94**	**[1.96–7.92]**	**3.70**	**[1.73–7.87]**	**3.75**	**[1.74–8.09]**
*Tablet*								
None	1143	10.1	1.00	reference	1.00	reference	1.00	reference
0.1–1 h/day	463	9.9	1.02	[0.71–1.47]	1.03	[0.70–1.51]	1.06	[0.71–1.56]
1.1–2 h/day	144	15.3	1.64	[1.00–2.68]	1.16	[0.68–1.99]	1.22	[0.70–2.10]
>2 h/day	87	29.9	**3.51**	**[2.08–5.91]**	**2.88**	**[1.63–5.09]**	**2.76**	**[1.52–5.01]**
*Portable game*								
None	1152	10.4	1.00	reference	1.00	reference	1.00	reference
0.1–1 h/day	536	9.7	0.89	[0.62–1.26]	1.00	[0.68–1.46]	1.01	[0.69–1.49]
1.1–2 h/day	108	23.1	**2.76**	**[1.69–4.51]**	**2.33**	**[1.29–4.20]**	**2.45**	**[1.34–4.49]**
>2 h/day	41	31.7	**3.78**	**[1.86–7.66]**	**2.87**	**[1.24–6.61]**	**2.86**	**[1.22–6.70]**
*Console game*								
None	1345	10.6	1.00	reference	1.00	reference	1.00	reference
0.1–1 h/day	398	11.8	1.16	[0.81–1.65]	1.43	[0.96–2.12]	1.42	[0.95–2.13]
1.1–2 h/day	77	16.9	1.68	[0.88–3.21]	1.26	[0.60–2.65]	1.27	[0.60–2.69]
>2 h/day	17	41.2	**6.55**	**[2.40–17.87]**	**4.04**	**[1.21–13.44]**	**4.69**	**[1.39–15.77]**
*TV/DVD*								
None	56	19.6	1.00	reference	1.00	reference	1.00	reference
0.1–1 h/day	354	8.2	**0.33**	**[0.15–0.72]**	**0.35**	**[0.16–0.81]**	**0.36**	**[0.16–0.84]**
1.1–2 h/day	666	9.3	**0.41**	**[0.20–0.84]**	**0.44**	**[0.20–0.96]**	0.45	[0.20–1.00]
>2 h/day	761	14.2	0.62	[0.31–1.25]	0.56	[0.26–1.22]	0.57	[0.26–1.26]
*Total screen time (4 devices excluding TV/DVD)*								
None	222	7.2	1.00	reference	1.00	reference	1.00	reference
0.1–1 h/day	861	7.9	1.10	[0.63–1.94]	1.08	[0.60–1.92]	1.09	[0.61–1.95]
1.1–2 h/day	417	13.4	**2.00**	**[1.12–3.57]**	**2.30**	**[1.25–4.24]**	**2.34**	**[1.26–4.35]**
2.1–3 h/day	146	12.3	1.81	[0.89–3.68]	1.74	[0.83–3.68]	1.73	[0.81–3.72]
3.1–4 h/day	94	19.1	**3.05**	**[1.48–6.28]**	**3.66**	**[1.69–7.90]**	**3.59**	**[1.63–7.91]**
>4 h/day	97	35.1	**6.95**	**[3.60–13.41]**	**8.57**	**[4.16–17.65]**	**8.37**	**[3.88–18.05]**

aOR: adjusted odds ratio; CI: confidence interval. Adjusted model included child’s sex, child’s age, presence of elder sibling, parents’ age, parents’ education, household income, mother’s occupation, screen media use within 1 h before sleeping, and child’s screen time of other devices (models for each device). Adjusted model 2 included parents’ screen time of mobile devices to Adjusted model. Bold numbers indicate *p* < 0.05.

**Table 4:** Associations between children’s delayed bedtime (after 22:00 h) and screen time on weekdays and weekends (N = 1837).

	Weekdays				Weekends			
Screen Time	n	% of cases	aOR	[95% CI]	n	% of cases	aOR	[95% CI]
*Smartphone*								
None	742	7.7	1.00	reference	657	6.8	1.00	reference
0.1–1 h/day	929	11.7	**1.56**	**[1.11–2.20]**	920	12.2	**1.83**	**[1.27–2.64]**
1.1–2 h/day	114	24.6	**3.85**	**[2.27–6.55]**	155	16.1	**2.50**	**[1.46–4.29]**
>2 h/day	52	30.8	**5.70**	**[2.84–11.45]**	105	26.7	**4.52**	**[2.58–7.90]**
*Tablet*								
None	1220	10.0	1.00	reference	1170	10.3	1.00	reference
0.1–1 h/day	403	10.4	1.01	[0.69–1.48]	372	9.9	0.92	[0.62–1.37]
1.1–2 h/day	140	15.7	1.43	[0.86–2.39]	143	11.9	1.04	[0.60–1.80]
>2 h/day	74	32.4	**3.72**	**[2.13–6.49]**	152	23.0	**2.08**	**[1.33–3.25]**
*Portable game*								
None	1253	10.5	1.00	reference	1174	10.2	1.00	reference
0.1–1 h/day	447	9.4	0.96	[0.65–1.41]	431	9.7	1.04	[0.70–1.53]
1.1–2 h/day	104	24.0	**3.06**	**[1.80–5.18]**	147	19.0	**2.46**	**[1.50–4.02]**
>2 h/day	33	33.3	**4.88**	**[2.18–10.94]**	85	23.5	**3.34**	**[1.85–6.04]**
*Console game*								
None	1463	10.9	1.00	reference	1360	10.5	1.00	reference
0.1–1 h/day	291	12.0	1.28	[0.85–1.93]	318	12.9	1.47	[0.99–2.17]
1.1–2 h/day	74	14.9	1.60	[0.80–3.20]	112	13.4	1.55	[0.85–2.83]
>2 h/day	9	55.6	**13.41**	**[3.19–56.34]**	47	23.4	**3.15**	**[1.49–6.66]**
*TV/DVD*								
None	83	13.3	1.00	reference	63	17.5	1.00	reference
0.1–1 h/day	383	9.1	0.59	[0.28–1.25]	224	6.7	**0.34**	**[0.14–0.80]**
1.1–2 h/day	673	9.1	0.61	[0.30–1.24]	485	9.5	0.53	[0.25–1.11]
>2 h/day	698	14.8	0.94	[0.46–1.93]	1065	13.0	0.63	[0.31–1.30]
*Total screen time (4 devices excluding TV/DVD)*								
None	375	7.2	1.00	reference	254	6.3	1.00	reference
0.1–1 h/day	863	9.3	1.32	[0.83–2.11]	760	8.3	1.31	[0.73–2.34]
1.1–2 h/day	339	11.2	**1.79**	**[1.04–3.08]**	366	11.7	**2.23**	**[1.19–4.15]**
2.1–3 h/day	124	15.3	**2.43**	**[1.25–4.71]**	184	14.1	**2.36**	**[1.19–4.67]**
3.1–4 h/day	66	24.2	**4.73**	**[2.26–9.88]**	110	17.3	**3.53**	**[1.68–7.43]**
>4 h/day	70	42.9	**12.09**	**[6.09–24.03]**	163	26.4	**5.94**	**[3.04–11.59]**

aOR: adjusted odds ratio; CI: confidence interval. The model included child’s sex, child’s age, presence of elder sibling, parents’ age, parents’ education, household income, mother’s occupation, screen media use within 1 h before sleep, and child’s screen time of other devices (models for each device). Bold letters indicate *p* < 0.05.

**Table 5:** Associations between children’s delayed bedtime (after 22:00 h) and types of Internet content accessed on smartphones or tablets among Internet users on the devices.

	Smartphone (n = 979)				Tablet (n = 623)			
	n	% of cases	aOR	[95% CI]	n	% of cases	aOR	[95% CI]
*Internet content accessed on the device ^1^ (reference: non-use of the content)*								
Video sharing	691	14.3	1.17	[0.76–1.81]	484	13.2	1.09	[0.60–2.00]
Game	499	17.4	**1.75**	**[1.16–2.65]**	274	17.2	**2.20**	**[1.31–3.69]**
Intellectual training	189	19.0	**1.65**	**[1.05–2.60]**	169	12.4	0.94	[0.53–1.66]
Music	130	14.6	0.94	[0.53–1.65]	63	6.3	0.37	[0.13–1.09]
Others	91	17.6	1.49	[0.80–2.77]	37	13.5	1.11	[0.39–3.15]
*Number of Internet contents accessed on the device*								
1	542	12.5	1.00	reference	339	12.7	1.00	reference
2	298	15.4	1.27	[0.83–1.94]	182	14.3	1.14	[0.66–1.99]
3 or more	139	20.9	**2.23**	**[1.34–3.70]**	102	14.7	1.51	[0.77–2.97]

aOR: adjusted odds ratio; CI: confidence interval. The model included child’s sex, child’s age, presence of elder sibling, parents’ age, parents’ education, household income, mother’s occupation, and screen media use within 1 h before sleeping. Bold numbers indicate *p* < 0.05. ^1^ The model also included all contents.

## Data Availability

Data are unsuitable for public deposition due to ethical restrictions and legal framework of Japan. It is prohibited by the Act on the Protection of Personal Information (Act No. 57 of 30 May 2003, amendment on 9 September 2015) to publicly deposit the data containing personal information. Ethical Guidelines for Medical and Health Research Involving Human Subjects enforced by the Japan Ministry of Education, Culture, Sports, Science and Technology and the Ministry of Health, Labour and Welfare also restricts the open sharing of epidemiologic data.

## Supplementary Materials

The following supporting information can be downloaded at: https://www.mdpi.com/article/10.3390/ijerph19159464/s1, Table S1: Comparison of the characteristics of children and parents between eligible respondents in the current study and other JECS participant mothers.
