# Supplementary material for: Association between Media Use and Bedtime Delays in Young Children: An Adjunct Study of the Japan Environment and Children’s Study
Source: Int J Environ Res Public Health. 2022 Aug 2;19(15):9464. doi: 10.3390/ijerph19159464 (PMC9368443; doi:10.3390/ijerph19159464)
Supplement: Supplementary file 1 [file ijerph-19-09464-s001.zip › ijerph-1815752-supplementary.pdf]

Table S1 Comparison of characteristics of children and parents between eligible respondents to the current study and other JECS participant mothers

	Eligible respondents		Other mothers		p-value
	n	%	n	%	
Number	1,837		3,231		
Basic characteristics					
Child's sex					
Male	940	51.2	1662	51.4	0.861
Female	897	48.8	1569	48.6	
Birth year					
2011	167	9.1	317	9.8	0.090
2012	629	34.2	1,112	34.4	
2013	617	33.6	1,149	35.6	
2014	424	23.1	653	20.2	
Elder sibling					
No	829	45.1	1,212	37.5	0.854
Yes	974	53.0	1,406	43.5	
Missing	34	1.9	613	19.0	
Mother's age at delivery, year mean (SD)	31.9	(4.7)	30.9	(5.1)	<0.001
Missing	0	0	1	0.03	
Father's age at 6 months, years mean (SD)	34.3	(5.9)	33.6	(6.0)	<0.001
Missing	55	3.0	372	11.5	
Mother's education					
Junior or senior high	559	30.4	1,226	37.9	<0.001
Junior college or vocational	801	43.6	1,215	37.6	
Undergraduate or above	454	24.7	541	16.7	
Missing	23	1.3	249	7.7	
Father's education					
Junior or senior high	712	38.8	1,458	45.1	<0.001
Junior college or vocational	444	24.2	712	22.0	
Undergraduate or above	656	35.7	792	24.5	
Missing	25	1.4	269	8.3	
Household income, million Japanese Yen					
<4	552	30.0	1,081	33.5	<0.001
4 to <6	631	34.3	985	30.5	

≥6	587	32.0	798	24.7	
Missing	67	3.6	367	11.4	
Mother's occupation at the age of 1 year					
No	1,037	56.5	1,599	49.5	0.737
Yes	760	41.4	1,197	37.0	
Missing	40	2.2	435	13.5	
Marital status at the age of 6 months					
Married	1,779	96.8	2,837	87.8	0.231
Single	29	1.6	61	1.9	
Missing	29	1.6	333	10.3	
Mother's media use while with her child at the age of 3 years, per day					
No	226	12.3	322	10.0	0.007
< 1 hour	1,153	62.8	1,508	46.7	
1 to < 2 hours	318	17.3	504	15.6	
≥ 2 hours	89	4.8	171	5.3	
Missing	51	2.8	726	22.5	
Child's media use at the age of 3 years, per day					
No	910	49.5	1,125	34.8	<0.001
< 1 hour	704	38.3	1,069	33.1	
1 to < 2 hours	136	7.4	231	7.1	
≥ 2 hours	39	2.1	84	2.6	
Missing	48	2.6	722	22.3	
Child's bedtime at the age of 3 years					
–19:59	183	10.0	201	6.2	0.087
20:00–20:59	622	33.9	863	26.7	
21:00–21:59	763	41.5	1,128	34.9	
22:00–22:59	182	9.9	246	7.6	
23:00–	29	1.6	51	1.6	
Missing	58	3.2	742	23.0	

SD, standard deviation
